# Fracture resistance, failure mode and stress concentration in a modified endocrown design

**DOI:** 10.1080/26415275.2020.1801348

**Published:** 2020-08-07

**Authors:** Wiam El Ghoul, Mutlu Özcan, Joao Paulo Mendes Tribst, Ziad Salameh

**Affiliations:** a Department of Prosthodontics, Faculty of Dental Medicine, Lebanese University, Beirut, Lebanon; b Division of Dental Biomaterials, Centre of Dental Medicine, Clinic of Reconstructive Dentistry, University of Zurich, Zurich, Switzerland; c Department of Dental Materials and Prosthodontics, São Paulo State University, Institute of Science and Technology, São José dos Campos, Brazil

**Keywords:** Endocrown, finite element analysis, lithium disilicate, fracture resistance, failure mode

## Abstract

**Purpose:**

The purpose of this study was to assess fracture resistance, failure mode and stress concentration of a modified endocrown preparation design, under axial and lateral forces.

**Materials and Methods:**

Forty lower molars were divided into two groups (*n* = 20) and were restored with lithium disilicate glass-ceramic endocrowns following 2 preparation designs: Conventional, with circumferential butt margin 2 mm above the cemento–enamel junction; and Modified, by adding 2 grooves on the mesial side of the vestibular dentinal wall and on the distal side of the lingual dentinal wall. After cementation and thermomechanical cycling loading, half of the samples (*n* = 10) from each group were loaded axially and the other half (*n* = 10) was loaded laterally. Fracture resistance and failure modes were observed and the finite element analysis (FEA) was used to identify the stress concentration. Two-way ANOVA and Chi-square tests (*α* = 0.05) were used for *in vitro* data analyzes.

**Results:**

Fracture resistance showed a statistically significant difference between conventional and modified preparations (*p* < .001), and between axial and lateral loadings (*p* < .001). Conventional preparation recorded 2914 N under axial loading and 1516 N under lateral loading, while modified preparation recorded 3329 N under axial loading and 1871 N under lateral loading. FEA showed that retention grooves have reduced the stress concentration under both loads for the tooth and the restoration.

**Conclusion:**

Modified endocrown design showed higher fracture resistance than conventional endocrown. Lateral loading displayed a high percentage of severe fracture but under higher load to failure than the values reported for normal masticatory forces.

## Introduction

Endodontically treated teeth (ETT) displayed a high risk of fracture caused by the extensive loss of dental tissue [1], which requires suitable restorations in order to repair the pulpless teeth saving their biomechanical properties [2,3]. Placement of metal posts improves retention of the crowns, but it increases the risk of a root perforation and root fracture [4,5]. The biomechanical properties of fiber posts are more comparable to that of dentine decreasing the risk of accidental root fractures [6], but building an adequate ferrule can necessitate crown lengthening leading to unfavorable crown to root ratio [7]. Developments in dental materials [8–10], computer-aided design/computer-aided manufacturing (CAD/CAM) technologies [11,12] and bonding systems [13,14] improve the application of endocrowns [15,16] to restore the ETT, that prevents the failures of posts [17,18]. Endocrown preparation preserves the dental structure, which has supragingival margins on peripheral enamel tissue enhancing adhesive capacity [19]. Endocrown restoration is more recommended in molars than premolars and anterior teeth because they have a small pulp chamber and they are more subjected to lateral forces [17,19]. The performance of endocrown is related to many factors such as types of material [10,20,21], axis of loading [10,22], and shapes of preparation [23–28]. Lithium disilicate glass ceramic (LDS) is the most common material for endocrown restorations [23,24,27] due to its biomechanical properties [29,30].

Depth and shape of the pulp chamber and form of the finish line (circumferential butt margin or chamfer finish line) are diverse tested preparation designs which can change the mechanical behavior of the endocrowns [14,23–28,31]: Tribst et al. showed in FEA study that stress is more concentrated on the restoration more than on the cement line if remaining dental tissue is bigger and if the material has high elastic modulus [26]. Hayes et al. [28] and Dartora et al. [25] displayed that the fracture resistance is superior with a deeper extension into the pulp chamber. While Zhu et al. presented that the central retainer form must follow the anatomical shape of the pulp chamber [24]. But De Kuijper et al. showed that extension of the endocrown core into the pulp chamber and type of outline don't significantly influence the fracture resistance of endocrown restorations [23]. Moreover, Ghajghouj et al. didn’t find a correlation between pulp chamber depth and fracture resistance values [14]. On the other side, Einhorn et al. and Taha et al. displayed that the addition of a shoulder finish line with short axial wall (ferrule design) can increase the fracture resistance of endocrown restoration more than circumferential butt margin design [27,31]. But no study tested the effect of grooves on the performance of endocrown restorations.

The aim of this *in vitro* study was to assess fracture resistance, stress concentration and failure mode of a new modified endocrown preparation design, and to compare the results with conventional endocrown preparation design, by using LDS ceramic, under lateral and axial forces. The two null hypotheses tested were (1) that design of preparation and type of loading would not affect fracture resistance and stress concentration, and (2) that design of preparation and type of loading would not affect the mode of failures.

## Methodology

**Figure 1.**
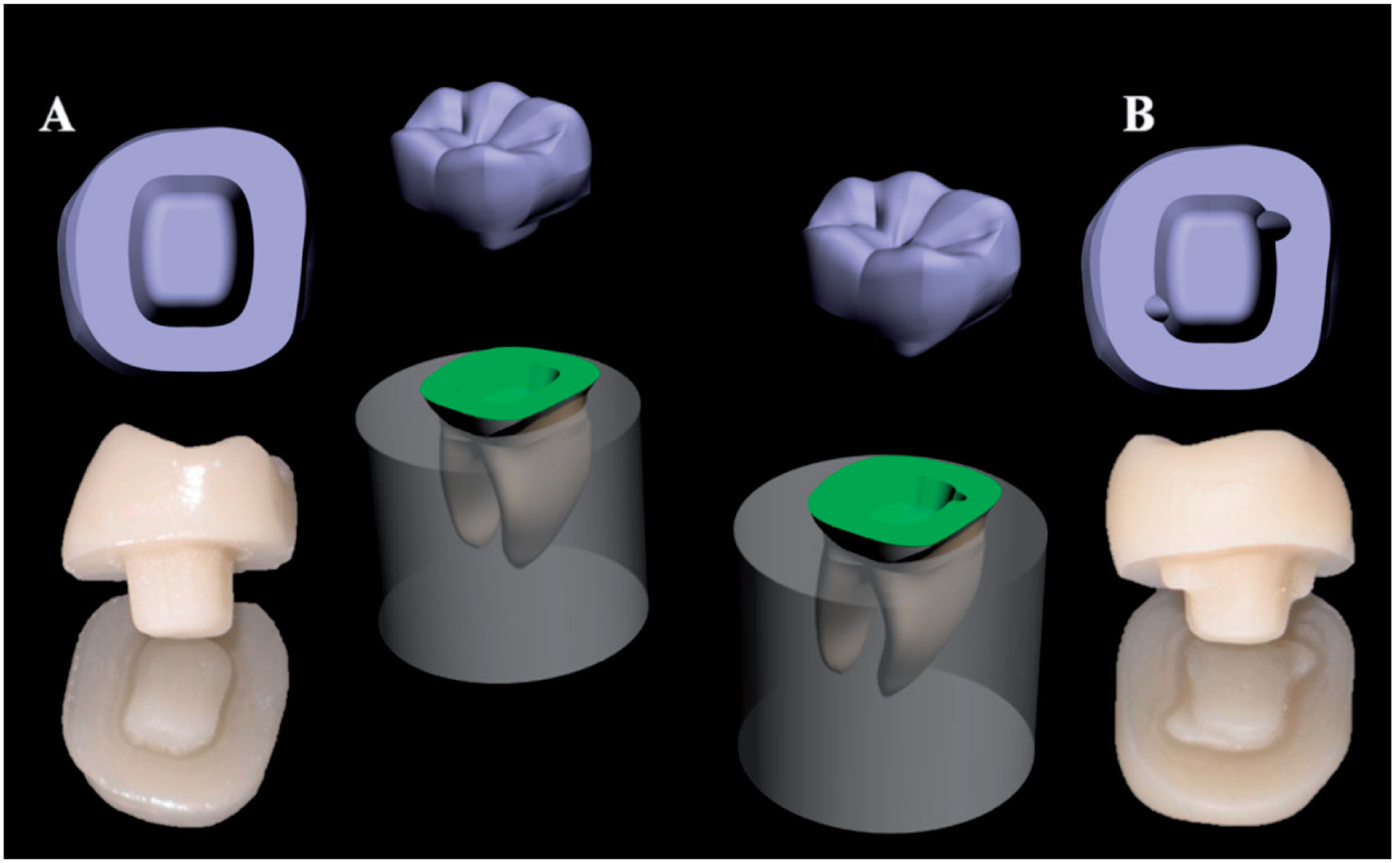
3D model and milled *in vitro* restorations: (A) conventional endocrown preparation design and (B) modified endocrown preparation design.

This study was accepted by an ethical committee of the Faculty of Dental Medicine, Lebanese University, Beirut, Lebanon (CUMEB/D123/102018). Forty sound human lower molars with almost comparable size and complete morphology were collected for this study. Teeth were conserved in a solution of chloramine 0.5% at 10 **°**C. After endodontic treatment using NiTi rotary instrumentation (Protaper Universal; Dentsply Sirona, New York, USA) and hot condenser system B (Sybron Endo; Henry Schein, Inc, Germany), all teeth were fixed parallel to their long axis in auto-polymerizing acrylic resin (Fastray; Harry J. Bosworth Co, Skokie, USA), utilizing dental surveyor (Marathon-103; Seayang, Korea) and metallic mold. The level of resin was 2 mm below cemento–enamel junction (CEJ) to imitate the level of bone. All teeth were prepared to have circular butt margin 2 mm above CEJ, conserving enamel tissue to get suitable bonding of the endocrowns [17]. Pulpal floors were covered with flowable resin composite (G-aenial Universal Flo, GC Corp). Final preparations were finalized applying a standardized method under water cooling using a dental lab parallel surveyor (Marathon-103; Seayang). Undercut areas were removed and pulpal floors were smoothed following the pulp chamber morphology, keeping a depth equal to 4 mm. Specimens were divided into two groups (*n* = 20) according to preparation designs: conventional endocrown design group, with circumferential butt margin 2 mm above the CEJ; and Modified endocrown design group by adding 2 grooves with a semi-circular shape (diameter = 2 mm), without sharp angles, same height of the dentinal wall (2 mm); one on the mesial side of the vestibular dentinal wall and one on the distal side of the lingual dentinal wall (Figure 1). All the internal angles were rounded.

Digital impressions were achieved utilizing an intraoral scanner (TRIOS 3; 3 Shape A/S, Germany). Captured data was kept as forty Standard Tessellation Language (STL) files and was transferred to a proper software (2017; 3 Shape Dental System, Germany) to construct identical restorations. Virtual endocrowns were milled in a 5-axis milling machine (Coritec 250i; imes-icore GmbH, Germany) from 14-size (IPS e.max CAD) blocks under wet processing. Finally, restorations were subjected to crystallization firing (Vita Vacumat, 6000 M, Vita Zahnfabrik GmbH, Germany) to get their final mechanical properties. All restorations were cemented according to the manufacturer's suggestions with a dual-cure resin adhesive (G-CEM LinkForce; GC Corp, Europe). Specimens were conserved in distilled water at 37 °C.

All specimens were subjected to thermomechanical cyclic loading using thermocycler **(**Willytec, Germany) and chewing simulator (CS − 4.2; Mechatronik GmbH, Germany). 3000 Thermocycles were done between 5 °C and 55 °C, while the settling time at every bath was 30 s and the transmission time between the 2 baths was 5 s. 300,000 loading cycles were made with a stainless-steel ball (diameter of 4 mm) in order to applicate force of 50 N on the center of the occlusal surface at the frequency of 1.6 Hz.

**Figure 2.**
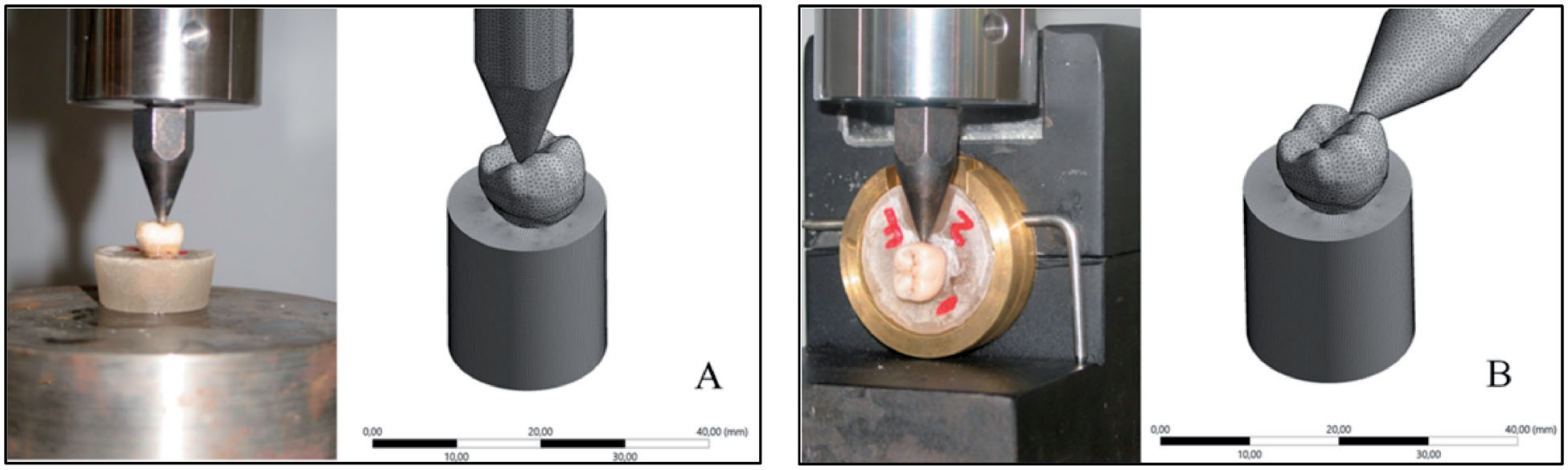
Compressive test using universal testing machine: (A) axial loading, (B) lateral loading for *in vitro* and in silico test respectively.

**Figure 3.**
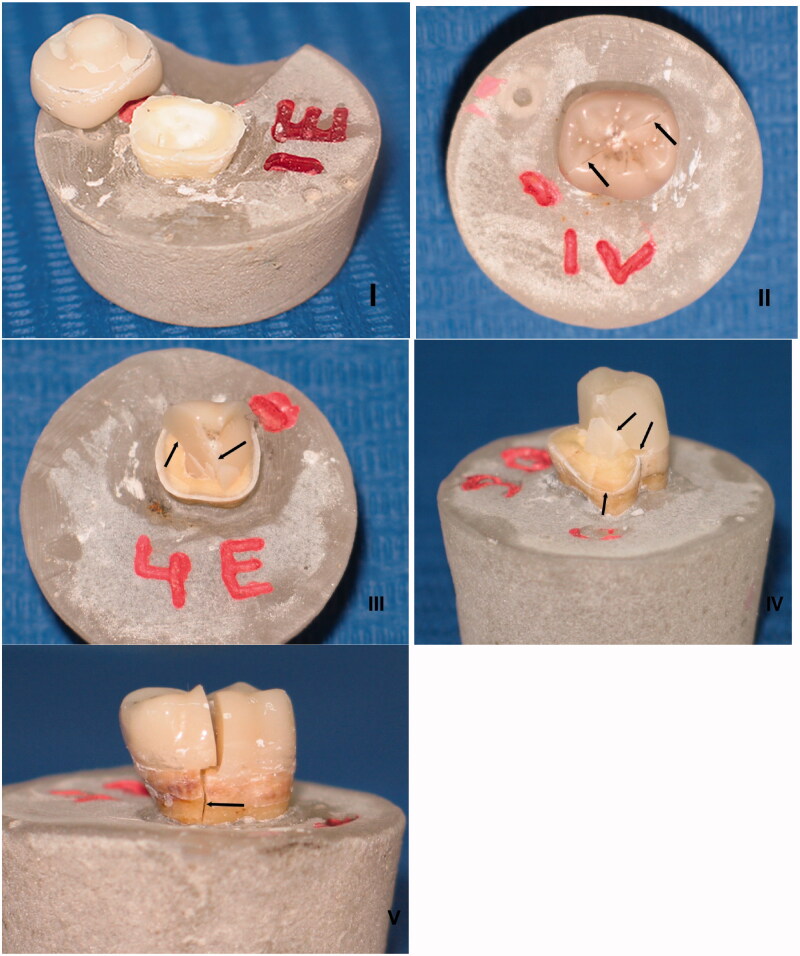
Type I: Cohesive failure; Type II: Adhesive failure; Type III: Cohesive-adhesive failure; Type IV: Fracture of the restoration/tooth complex above the cemento-enamel junction (CEJ); Type V: Fracture of the restoration/tooth complex below the cemento-enamel junction (CEJ).

Fracture resistance test was accomplished employing a universal testing machine (Treviolo; Matest Spa, Italy) with a stainless-steel ball (diameter of 3 mm) at a cross-head speed of 0.5 mm/minute. Specimens were divided into four groups (*n* = 10) according to loading directions, which was adequate for a statistical power of 80% (G*Power 3.1.9.2.) [25,28]. Half of the specimens from every preparation design was loaded vertically on the center of the occlusal surface (axial loading). While the other half was loaded laterally next to the border endocrown-tooth, perpendicular to the tooth long axis (lateral loading) [22,32] (Figure 2). Samples were fixed in a metallic device to prevent rotation. Load at fracture was recorded in newton (N). All samples were analyzed using a stereomicroscope (Amscope 3.5; Irvine, USA) and a scanning electron microscope SEM (AIS 2100; Seron technologies, Inc, Korea) in order to define the mode of failures and to establish a fractographic analysis based on the principles of fractography [33]. The mode of failures was described according to a classification summarized in Table 1 and Figure 3.

**Table 1.** Classification of the failure modes.

Type	Failure mode	Description
I	Cohesive failure	Fracture of the endocrown without displacement (loss of adhesion)
II	Adhesive failure	Debonding of the endocrown without fracture
III	Cohesive-adhesive failure	Fracture of the endocrown with displacement (loss of adhesion)
IV	Fracture of the restoration/tooth complex above CEJ	Fracture of the endocrown and the tooth above CEJ
V	Fracture of the restoration/tooth complex below CEJ	Fracture of the endocrown or/and the tooth below CEJ, which require tooth extraction

CEJ: Cemento-enamel Junction.

A numerical simulation by finite element was used for identifying the stress concentration in the system. A conventional endocrown restoration on molar was exported to the modeling software (Rhinoceros 5.0 – SR9 McNeil, North America) and was completed in order to obtain the conventional design 3 D model [26]. Then, this 3D model was duplicated and the replica was isolated to have an experimental design group. Following the *in vitro* samples, this new model received two pulp chamber grooves. A Boolean union was used to create a single volumetric endocrown restoration with a positive model of the grooves integrated in its structure. In the sequence, dental model and cement layer have received a boolean split with the cutting object determined by the new endocrown design. Finally, restoration, tooth and cement were individualized according to the presence of grooves in the pulp chamber. Conventional and modified endocrown design geometries were subsequently exported to finite element analysis software (ANSYS 17.2, ANSYS Inc., Houston, TX, USA). Mechanical properties of the materials (elastic modulus and Poisson’s ratio) were attributed according to the literature. Simulated materials were: LDS restorations [IPS e.max CAD, elastic modulus (*E*) = 82.3 GPa and Poisson ratio (*ν*) = 0.22] [34], sound dental remnant (Enamel, *E* = 84.1 GPa and *ν* = 0.33; Dentin, *E* = 18.6 GPa and *ν* = 0.31), and uniform composite resin cement (*E* = 8.2 GPa and *ν* = 0.30) [35]. A static mechanical analysis was performed within the elastic limit, in which all geometries were composed of homogeneous materials with linear and isotropic behavior [34]. The mesh convergence test was assisted to determine the meshing for each model (Conventional model with 198,653 nodes and 96,484 tetrahedral elements, modified model with 212,472 nodes and 108,648 tetrahedral elements). Aspect ratio of the mesh elements presented an average of 1.8 ± 0.7.

**Figure 4.**
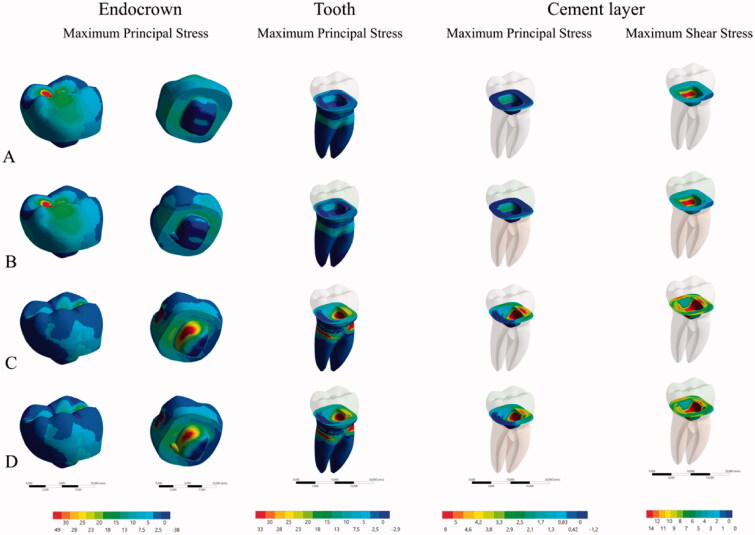
Stress maps according to each group: (A) in the conventional endocrown restorations with axial loading, (B) in the conventional endocrown restorations with lateral loading, (C) in the modified endocrown design with axial loading and (D) in the modified endocrown design with lateral loading.

**Figure 5.**
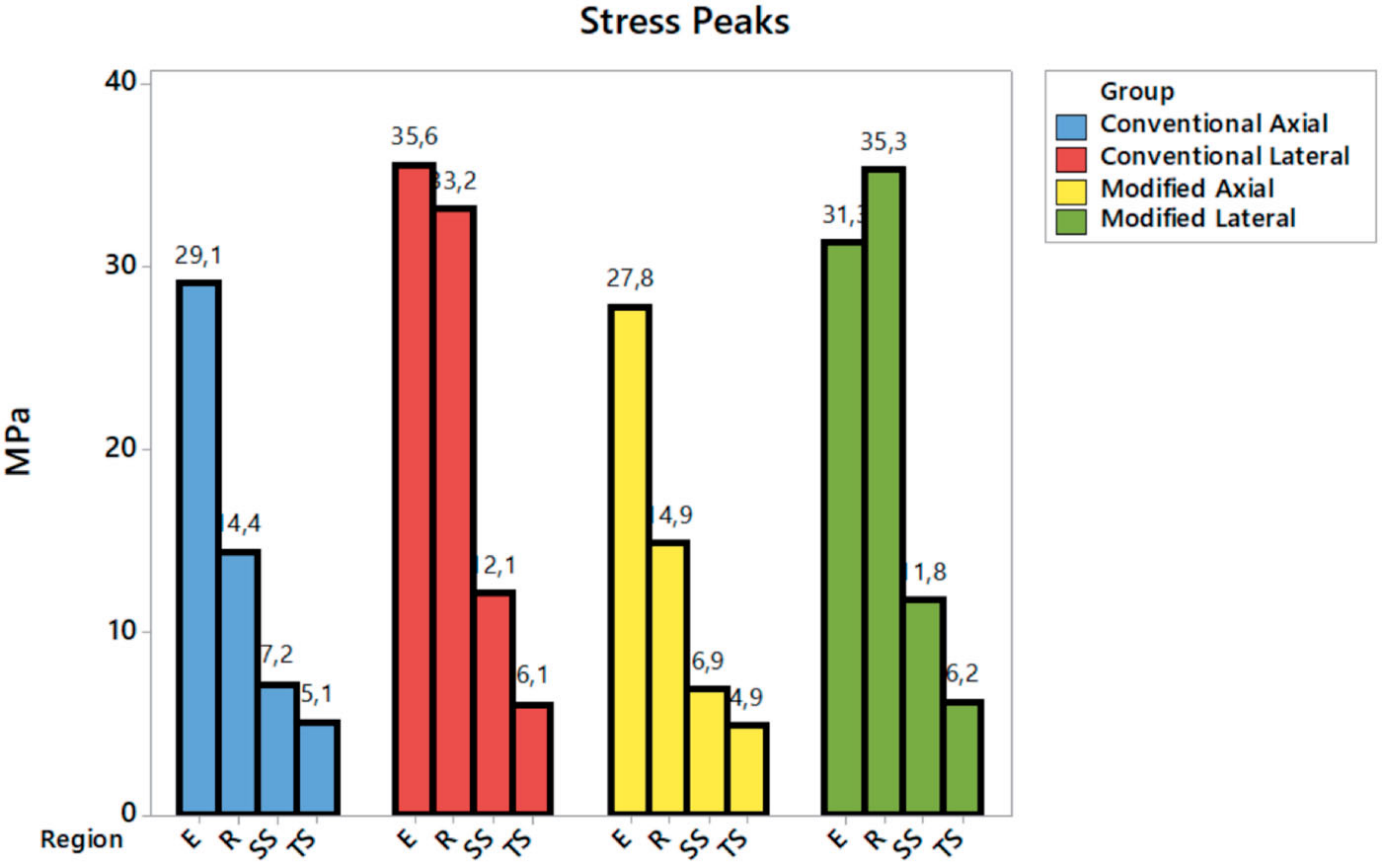
Tensile stress peaks recorded for each structure during the four different simulations. E: Endocrown tensile stress; R: Root dentin tensile stress; SS: Cement shear stress; TS: Cement tensile stress.

For the boundary conditions, *in vitro* test was used to determine the loading and the fixations (1500 N). Set was fixed by the base of the acrylic resin cylinder with fixed zero nodal displacements. Load was applied within two different simulations (Figure 2). Results were obtained using maximum principal stress criteria for restoration and tooth. Maximum principal stress and maximum principal shear stress criteria were recorded for the cement layer (Figure 4). Stress distribution was plotted in colorimetric maps and stress peaks in each structure are summarized in Figure 5.

Descriptive statistics for load to failure and for failure mode were generated by preparation design and by loading type. Load to failure outcomes were normally distributed (Shapiro-Wilk’s *p* value > .05). A two-way ANOVA test was used in order to compare mean load to failure between the two preparation designs by loading type. The 2-way ANOVA test was followed by reporting of main effects for design and for loading type. Chi-square tests were used for testing the association between failure mode and each of preparation design and loading.

Data are presented as mean ± standard deviation. The IBM^®^ SPSS^®^ statistics 20.0 statistical package and Stata MP/13.0 were used to carry out all statistical analyses. Statistical significance was set at 0.05. The stress peaks were quantitatively compared and values with more than 10% (Based in mesh convergence test) of difference assumed as significant.

## Results

Mean load to failure levels ranged between 1516 ± 202 N for the conventional design group under lateral loading and 3329 ± 134 N for the modified design group under axial loading (Table 2). There was no statistically significant interaction between preparation design and loading type (*p* = .570). The main effects for loading and for preparation design were statistically significant (*p* < .001; Table 3). Mean of the load to failure was statistically significantly larger in the modified design group than in the conventional design group by a difference of 385 ± 53 N (*p* < .001). Mean of the load to failure was also statistically significantly larger under axial loading than under lateral loading by a difference of 1429 ± 53 N (*p* < .001; Table 3).

**Table 2.** Distribution of load (*N*) to failure by preparation design and loading type (*n* = 40).

					95% confidence interval	
	Mean	SD	Minimum	Maximum	Lower bound	Upper bound
Conventional preparation design						
Axial	2914	205	2607	3278	2768	3061
Lateral	1516	202	1235	1794	1371	1660
Modified Preparation design						
Axial	3329	134	3098	3509	3233	3425
Lateral	1871	99	1725	2054	1800	1941

**Table 3.** Distribution of load (*N*) to failure by 2-way ANOVA main effects of preparation design and loading (*n* = 40).

			Difference			
	Mean	SE	Mean	SE	*F*	*p*
Main effects of design						
Conventional	2215	37	−385	53	53.481	<.001**
Modified	2600	37				
Main effects of loading						
Axial	3122	37	1429	53	736.865	<.001**
Lateral	1693	37				

No statistically significant interaction present between preparation design and loading (*p* = .570).

**Statistically significant, *p* < .001; SE: standard Error.

Overall, the majority of failures were type V (Table 4). In the conventional preparation design group, 50% and 60% of failures were type V under axial and lateral loading, respectively. Under axial loading, type III failures displayed a percentage of 40%. But under lateral loading, all types I, II, III and IV displayed an equal percentage of 10%. In the modified preparation design group, 80% of failures were type V under both axial and lateral loading. 20% of failures only were distributed between type I and type III under axial loading, and among type II and type III under lateral loading (Table 4). There was no statistically significant association between failure mode and preparation design (Chi-square = 2.849; *p* = .091). There was similarly no statistically significant association between failure mode and loading (Chi-square = 0.114; *p* = .736).

**Table 4.** Distribution of failure modes by groups and loading type (*n* = 40).

Failure mode *n* (%)					
	I	II	III	IV	V
Conventional preparation					
Axial	1 (10)	0 (0)	4 (40)	0 (0)	5 (50)
Lateral	1 (10)	1 (10)	1 (10)	1 (10)	6 (60)
Modified preparation					
Axial	1 (10)	0 (0)	1 (10)	0 (0)	8 (80)
Lateral	0 (0)	1 (10)	1 (10)	0 (0)	8 (80)

Type I: Cohesive failure; Type II: Adhesive failure; Type III: Cohesive-adhesive failure; Type IV: Fracture of the restoration/tooth complex above the cemento-enamel junction (CEJ); Type V: Fracture of the restoration/tooth complex below the cemento-enamel junction (CEJ).

**Figure 6.**
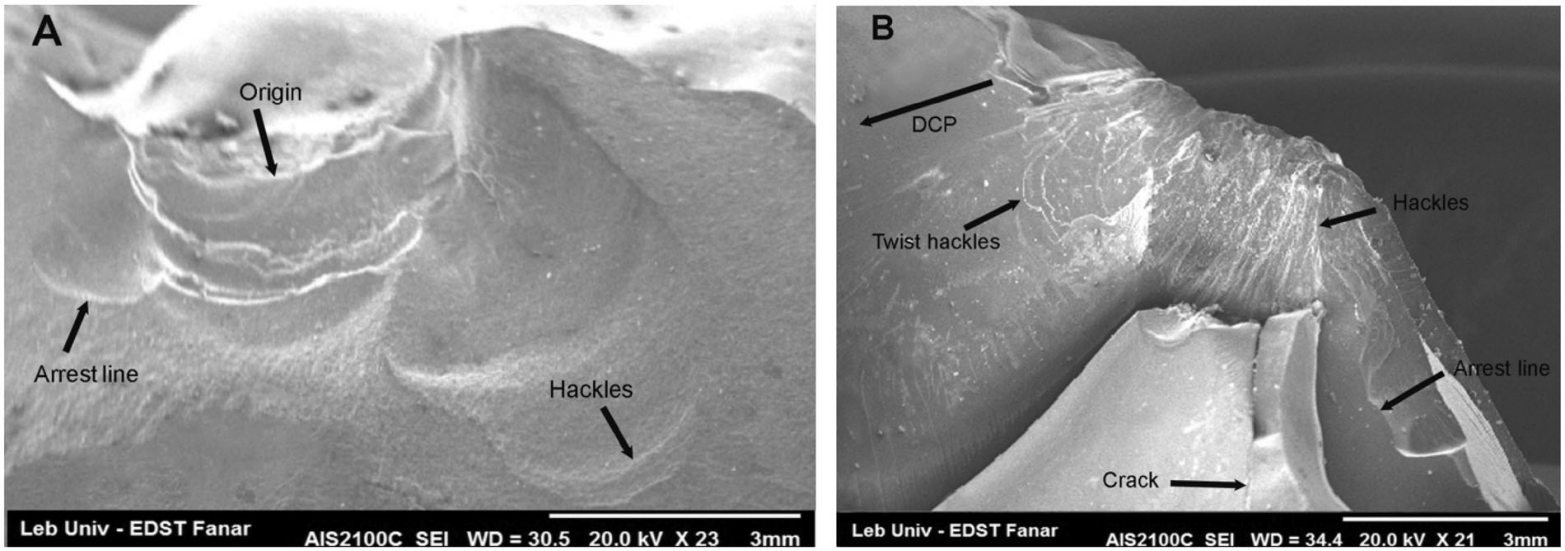
Features of fractographic analysis.

The fractographic analysis showed that the fracture origin was at the point of loading in all fractured specimens. The fracture propagated corono-apically toward the long axis under the axial loading, but the direction of crack propagation was laterally toward the cervical area under lateral loading. Fracture markings identified were hackles, wake hackles, arrest lines and direction of crack propagation (DCP) (Figure 6).

Higher tensile stress concentration occurs in the restoration intaglio surface regardless of loading and design (Figure 4). Conventional design showed more stress concentration in the pulp chamber extension during lateral loading, this same behavior was noted for the modified design model but with reduced magnitude (35.6–31.3 MPa) (Figure 5). The presence of retention grooves in the modified design reduced the stress concentration in the restoration for both performed loads (11% in the axial and 4.34% in the lateral).

For the cement layer, maximum shear stress was calculated during lateral loading for both designs (12.1 MPa for conventional and 11.8 MPa for modified design) (Figure 5). The tensile stress concentration in the cement layer was reduced with the use of the modified design but the highest peak was only 11.25% of the shear stress peak.

Stress distribution in root dentin was approximately similar between the two designs, but the highest stress peak was calculated under lateral loading (Figure 5).

## Discussion

Results supported the refusal of the first null hypothesis that type of preparation and type of loading would not affect load to failure and acceptance of the second null hypothesis that type of preparation and type of loading would not affect failure modes.

Fracture resistance showed a statistically significant difference between both designs (*p* < .001) (Table 3), which suggests that these grooves increase the retention of micro-mechanically and macro-mechanically. The adhesive surface between abutment teeth and endocrowns was extended (micro-mechanical retention), enhancing the transmission of forces to dental abutments. Moreover, these grooves may engage lingual and buccal pulpal walls, improving the macro-mechanical retention by cross-section shape, which results in increasing the endocrown’s fracture resistance [36]. This outcome corroborates with other previous studies that examined the effect of increasing the bonding surface between abutment teeth and endocrowns by increasing pulp chamber depths: Dartora et al. showed that deeper pulp chamber (5 mm) recorded higher load to failure and reduced stress concentration in comparison with reduced pulp chamber depth (1 mm) using LDS material [25]; Hayes et al. showed that 4 mm pulp chamber depth presented higher failure load than 2 mm, using LDS [28].

The load was applied in 2 directions (axial and lateral) to replicate all occlusal forces (compression and shear stresses) and to test the adhesion competence of LDS endocrown [10,22]. Recorded values displayed a statistically significant difference between axial and lateral loadings (*p* < .001), those were above the human masticatory forces ranging from 200 to 900 N, while lateral forces varied entirely in the range of 200 N [37]. The results in this study corroborate with Gresnigt et al. and El Ghoul et al., who showed that load to failure under axial loading is higher than under lateral loading using endocrowns in LDS [10,22]. The authors justified this effect because lateral loading doesn't distribute stress along the long axis of the abutment teeth and concentrates pressure in the cervical areas [22,38]. Forces towards the teeth/restorations are multidirectional and the results from this *in vitro* study indicate that restorations under lateral loading displayed severe fractures; however, with a higher load to failure than the reported normal values of masticatory forces, as well as parafunctions [37]. These results corroborate with previous studies of Hayes et al., Altier et al. and De Kuijper et al. that related this result with the LDS rigidity and high elastic modulus (95 GPa) [20,23,28], concentrating more stresses in weak regions, leading to catastrophic failures [39]. Deeper extension into the pulp chamber increases bonding surfaces and load to failure values, however transmits all the forces to the root dentin[28]. And the lateral loading concentrates strain in the cervical area and does not distribute it toward the long axis running to non-restorable fractures [38].

The fractographic analysis revealed that the main crack started on the external surface of the restoration near the loading point and propagated inside the restoration axially or laterally depending on the loading axis. The fractures could involve the dental tissue throughout the restoration/tooth interface leading to catastrophic failures because of the strong adhesion of the LDS to the enamel tissue [29,30]. The SEM images revealed high proportions of cracks around the loading ball that might be influenced by the aging process [25].

Observing finite element results, the stress maps herein (Figure 4) corroborated with a previous paper [26] which showed that a higher stress concentration occurred on the intaglio surface of the endocrown. Results suggest that retention grooves in the pulp chamber walls can improve the load to fracture and stress concentration. Lateral loading showed higher stress concentration in the cement layer, thus it is possible to assume that failures in the cement layer can occur and will be facilitated by lateral loading due to a higher concentration of shear forces (Figure 4). These results corroborate with the *in vitro* results (Table 2). Lateral loading generated failures regardless of the design. It was already reported for premolar endocrowns and could explain why it is so important for the clinician to evaluate the tooth position prior performing this restorative modality [40]. Although the incidence of oblique loads in molars is not as common as the loads incident on premolars, the results showed that they could be more damaging than axial loads for molars restored with endocrowns also (Figure 5).

The limitations of this study is that it was an *in vitro* study that cannot simulate all oral conditions. Materials simulated in FEA are isotropic and homogeneous, with a uniform cement layer without any bubbles or defects. Further *in vivo* studies are required, using additional materials and CAD/CAM systems with different preparation shapes in order to test the clinical performance of the endocrown restorations. Despite this, the results herein are valid since the in silico and *in vitro* tests corroborated in the presented mechanical behavior.

## Conclusions

From this study, the following conclusions could be drawn:

- Modified endocrown preparation design (with grooves) showed a higher fracture resistance and a reduced stress concentration in comparison with conventional endocrown preparation design.
- Majority of failures were severe, especially in the modified endocrown design (80%); however, this design showed a higher load to failure than the values reported for normal masticatory forces, as well as parafunction.
